# Roles of the Fungal-Specific Lysine Biosynthetic Pathway in the Nematode-Trapping Fungus *Arthrobotrys oligospora* Identified through Metabolomics Analyses

**DOI:** 10.3390/jof9020206

**Published:** 2023-02-05

**Authors:** Hengqian Lu, Shuai Wang, Tiantian Gu, Liangyin Sun, Yongzhong Wang

**Affiliations:** 1 School of Life Sciences, Anhui University, Hefei 230601, China; 2 Key Laboratory of Human Microenvironment and Precision Medicine of Anhui Higher Education Institutes, Anhui University, Hefei 230601, China; 3 Anhui Key Laboratory of Modern Biomanufacturing, Hefei 230601, China

**Keywords:** predatory nematode fungi, *Arthrobotrys oligospora*, metabolomics, α-aminoadipic acid reductases, lysine metabolism, non-ribosomal peptides

## Abstract

In higher fungi, lysine is biosynthesized via the α-aminoadipate (AAA) pathway, which differs from plants, bacteria, and lower fungi. The differences offer a unique opportunity to develop a molecular regulatory strategy for the biological control of plant parasitic nematodes, based on nematode-trapping fungi. In this study, in the nematode-trapping fungus model *Arthrobotrys oligospora*, we characterized the core gene in the AAA pathway, encoding α-aminoadipate reductase (*Aoaar*), via sequence analyses and through comparing the growth, and biochemical and global metabolic profiles of the wild-type and *Aoaar* knockout strains. *Aoaar* not only has α-aminoadipic acid reductase activity, which serves fungal L-lysine biosynthesis, but it also is a core gene of the non-ribosomal peptides biosynthetic gene cluster. Compared with WT, the growth rate, conidial production, number of predation rings formed, and nematode feeding rate of the Δ*Aoaar* strain were decreased by 40–60%, 36%, 32%, and 52%, respectively. Amino acid metabolism, the biosynthesis of peptides and analogues, phenylpropanoid and polyketide biosynthesis, and lipid metabolism and carbon metabolism were metabolically reprogrammed in the Δ*Aoaar* strains. The disruption of *Aoaar* perturbed the biosynthesis of intermediates in the lysine metabolism pathway, then reprogrammed amino acid and amino acid-related secondary metabolism, and finally, it impeded the growth and nematocidal ability of *A. oligospora*. This study provides an important reference for uncovering the role of amino acid-related primary and secondary metabolism in nematode capture by nematode-trapping fungi, and confirms the feasibility of *Aoarr* as a molecular target to regulate nematode-trapping fungi to biocontrol nematodes.

## 1. Introduction

The biological control of plant parasitic nematodes has the potential to replace or to reduce the usage of environmentally unfriendly chemical insecticides [1,2]. Nematode-trapping fungi are the natural enemies of plant parasitic nematodes. More than 700 species of these fungi are able to prey on living nematodes and to use them as a nutrient source [3,4,5]. The nematodes induce a switch in the lifestyle of the nematode-trapping fungi, from a saprophytic to a parasitic stage. In response, the fungi form specific trapping structures such as adhesive networks, adhesive knobs, and constricting rings to capture nematodes and to digest them [6,7,8]. Recently, with the development of omic technologies, several nematode-trapping fungi have been sequenced, and the mechanisms involved in the lifestyle switch have begun to be understood through multi-omics analyses at a system level [7,9]. These studies have provided a broad basis for studying the regulatory mechanisms of hyphal growth and development, cell differentiation, and predacious ability in nematode-trapping fungi [10,11,12,13].

Amino acid metabolism has been revealed to be critical in the processes of hyphal growth, development, and trap formation in nematode-trapping fungi. However, amino acid metabolic pathways are complex and they involve many genes. Further studies focusing on individual gene knockouts will be necessary to determine the roles of these genes in amino acid metabolism during hyphal growth and trap formation. Lysine biosynthesis is one of the ways in which higher fungi differ biochemically from other species [14]. Higher fungi use the α-aminoadipic acid pathway to synthesize lysine, unlike bacteria and plants, which use the diaminoadipic acid pathway, and humans, who cannot synthesize lysine. The α-aminoadipate pathway of lysine biosynthesis therefore offers a unique opportunity for the control of microbial consortia to obtain growth advantages for nematode-trapping fungi in complex soil microhabitats, which are characterized by multiple species [15,16,17]. However, the genes and enzymes of this pathway have not been investigated in any nematode-trapping fungi. Although the conversion of α-aminoadipic acid to α-aminoadipic-semialdehyde catalyzed by α-aminoadipate reductase (AAR) is an obligatory step for the biosynthesis of lysine in fungi [18], the characteristics of AAR in nematode-trapping fungi are poorly understood.

*Arthrobotrys oligospora* has been extensively studied as the model species for nematode-trapping fungi [7,19]. As with other nematode-trapping fungi, nematodes can induce *A. oligospora* to switch from a saprophytic stage to a parasitic stage. In the parasitic stage, *A. oligospora* produces many 3D predation rings, which then attract, bind, capture, penetrate, and digest nematodes, which then provide nutrients for the growth of the fungus.

In this study, the putative gene *AOL_s00081g219*, coding α-aminoadipate reductase (*Aoaar*) in *A. oligospora*, was identified. By comparing the growth, and biochemical and global metabolic profiles of wild-type and *Aoaar* knockout strains, the functions of *Aoaar* were characterized.

## 2. Materials and Methods

### 2.1. Strains, Plasmids, and Culture Conditions

*Arthrobotrys oligospora* ATCC 24,927 was purchased from the American Type Culture Collection (Manassas, VA, USA). *Arthrobotrys oligospora* ATCC 24,927 was cultured in TYGA medium (10 g/L tryptone, 10 g/L dextrose, 5 g/L yeast extract, 5 g/L molasses, and 15 g/L agar) at 28 °C for mycelial culture. Conidia were obtained from *A. oligospora* grown in corn meal agar (CMA) plates at 28 °C for 10 days [20]. *Caenorhabditis elegans*, which was stored in our laboratory, was cultured in NGM medium (3 g/L NaCl, 2.5 g/L peptone, 17 g/L agar, 5 mg/L cholesterol-ethanol solution, 1 mM MgSO_4_, 1 mM CaCl_2_, and 12.5 mM KH_2_PO_4_-K_2_HPO_4_ solution) containing *Escherichia coli* OP50 for 7 d at 20 °C. *Caenorhabditis elegans* were collected by washing NGM plates with M9 buffer solution (22 mM KH_2_PO_4_, 42 mM Na_2_HPO_4_, 85.5 mM NaCl, and 1 mM MgSO_4_) [21]. The plasmids pUC19 and pUC57–hygR were maintained in *E. coli* DH5α.

### 2.2. Sequence Information Analysis

The nucleic acid and predicted amino acid sequences of *AOL_s00081g219* (*Aoaar*) were searched for and downloaded from NCBI. The online program protein BLAST (Blastp) was used to analyze the conserved domains in the AoAAR. The phylogenetic tree of *Aoaar* genes was constructed using MEGA 7.0 software [22]. The prediction of secondary metabolite biosynthetic gene clusters was performed using AntiSMASH online software (https://antismash.secondarymetabolites.org/#!/start, accessed on 27 December 2022) [23].

### 2.3. Construction of the Aoaar Disruption Vector

The construction of the *Aoaar* disruption vector was undertaken using similar methods as in our previous report [21]. Genomic DNA was extracted using a fungal DNA extraction kit (Solarbio, Beijing, China). Using *Aoaar*-5F/*Aoaar*-5R and *Aoaar*-3F/*Aoaar*-3R primer pairs (Table S1), the 5′ and 3′ flanking sequences of *Aoaar* were amplified from the genomic DNA and marked as *Aoaar*-up and *Aoaar*-down. The hygromycin resistance gene (*hygR*) cassette was amplified from the pUC57–*hygR* vector with the primers *Hph*-F and *Hph*-R (Table S1), and designated *hygR*. The DNA amplicons were amplified via PCR using high-fidelity KOD-Plus DNA polymerase (TOYOBO, Osaka, Japan). The plasmid pUC19 was digested with *NdeI* and *PciI* (Transgen Biotech, Beijing, China) restriction enzymes to prepare the linearized pUC19 vector. The three amplicons, *Aoaar*-up, *hygR*, and *Aoaar*-down, were ligated with the linearized pUC19 vector using an In-Fusion^®^ HD Cloning Kit (Takara Bio, San Jose, CA, USA) to generate the knockout plasmid Δ*Aoaar*. The knockout plasmid was transformed into *E. coli* DH5α, and the transformants were selected by screening on LB plates containing ampicillin. Plasmids were verified through DNA sequencing.

### 2.4. Transformation of Protoplasts

The transformation of protoplasts was according to our previously described methods [21,24]. The knockout DNA fragment was amplified from the knockout plasmid using the *Aoaar*-5F and *Aoaar*-3R primer pair. The linear knockout DNA fragments were then transformed into *A. oligospora* via protoplast transformation to knock out *Aoaar*. To prepare the protoplasts, the WT strain of *A. oligospora* was cultured onto Potato Dextrose Agar (PDA) plates (200 g/L potato infusion, 20 g/L dextrose, and 15 g/L agar) for 7 d, then transferred to TG medium (10 g/L tryptone and 10 g/L dextrose) and incubated statically for 16 h at 28 °C, followed by shaking at 160 rpm and 28 °C for 24 h. The mycelia were collected via filtration and resuspended in MN buffer (0.3 M MgSO_4_, 0.3 M NaCl, 7.5 g/L cellulase, and 5 g/L snail enzyme). The suspension liquid was then incubated at 30 °C with shaking at 180 rpm for 4 h. After enzymatic hydrolysis, the solution was filtered and centrifuged at 4500× *g* for 10 min to collect the pellets for the protoplast preparation. The protoplast pellets were washed twice with KTC buffer (1.2 M KCl, 10 mM Tris-HCl, and 50 mM CaCl_2_), and finally resuspended in KTC buffer. The protoplasts (100 µL) were then mixed with 10 µg linearized DNA fragments and maintained in an ice bath for 40 min, followed by the addition of 700 µL PTC buffer (50 mM CaCl_2_, 20 mM Tris-HCl, and 60% polyethylene glycol 6000; pH 7.5) and incubated at 28 °C for 1 h. The mixture was then spread on non-selective TB3 plates (200 g/L sucrose, 3 g/L tryptone, 3 g/L yeast extract, and 15 g/L agar). After incubating for 18 h, an upper layer of TB3 agar supplemented with 200 μg/mL hygromycin B and 7.5 g/L agar was poured on the plates, and the plates were cultured at 28 °C for 7 d. The transformants were transferred to TYGA plates, which are non-selective. The mycelia were finally collected for PCR analysis to verify the positive transformants.

### 2.5. Comparison of Mycelial Growth and Morphology

The WT and Δ*Aoaar* mutant strains were inoculated on PDA plates and cultured at 28 °C for 7 d for activation. Then, mycelial disks 1 cm in diameter were cut from the plates and inoculated into PDA, TYGA, and CMA plates and cultured at 28 °C with 50% humidity in an incubator. Colony morphologies were observed, and their diameters were measured every 24 h [20,24]. From these measurements, the growth rates were calculated.

### 2.6. Comparison of Conidial Production, Morphology, and Germination

The WT and Δ*Aoaar* mutant strains were cultured in PDA plates for 7 d to ensure that there was active growth. Then, mycelial disks 1 cm in diameter were cut from the plates, inoculated at the centers of the CMA plates, and cultured at 28 °C for 7 d. The conidia were then rinsed from the plates using 20 mL sterile ddH_2_O, followed by filtration through four layers of lens cleaning paper, and centrifugation at 4000× *g* for 10 min. The conidia were counted via microscopy on a hemocytometer. These conidial suspensions were diluted to a concentration of 1 × 10^5^ conidia/mL, and then 20 µL was inoculated on water agar (WA) plates (15 g/L agar) to record the conidial germination rates every hour [20,24].

### 2.7. Trap Formation and Nematicide Activity

Approximately 2 × 10^3^ conidia of the WT and Δ*Aoaar* mutant strains were spread on WA medium and germinated at 28 °C for 72 h. Subsequently, approximately 500 *C. elegans* nematodes were added to the middle of each plate to induce trap formation. The numbers of traps induced and nematodes captured were counted under a light microscope every six hours for 24 h [7,20,21].

### 2.8. Metabolomics Analysis

Mycelial samples stored at −80 °C were thawed at room temperature. Mycelium (80 mg) was added to a 1.5 mL Eppendorf tube with 20 μL of L-2-chlorophenylalanine (0.06 mg/mL) dissolved in methanol as an internal standard, along with two small steel balls. The tube was vortexed for 10 s. Subsequently, 700 μL of an ice-cold mixture of methanol-water (7:3, *v*/*v*) was added, and the mixtures were vortexed for 1 min and then added to a grinder (60 Hz, 2 min) after pre-cooling at −20 °C for 2 min. The samples were extracted through ultrasonication in an ice-water bath for 30 min, and then stored at −20 °C overnight. The samples were centrifuged at 4 °C (13,000 rpm) for 10 min. The supernatants (150 μL) from each tube were collected using crystal syringes, filtered through 0.22 μm microfilters, and transferred to LC vials. The vials were stored at −80 °C until liquid chromatography–mass spectrometry (LC-MS) analysis. Samples for quality control (QC) were prepared by mixing aliquots of all samples to a pooled sample [9,25].

The untargeted metabolomics analysis was conducted using a Dionex UltiMate 3000 UPLC system (Santa Clara, CA, USA) coupled with a HESI probe with a Q Exactive Orbitrap mass spectrometer (Thermo Fisher Scientific, Sunnyvale, CA, USA) [26]. The metabolites extracted from *A. oligospora* were separated on an ACQUITY UPLC HSS T3 column (1.8 μm, 2.1 × 100 mm) maintained at 40 °C. The binary gradient elution system consisted of (A) water (containing 0.1% formic acid, *v*/*v*) and (B) acetonitrile (containing 0.1% formic acid, *v*/*v*), and separation was achieved using the following gradient: 0.01 min, 5% B; 1 min, 5% B; 2.5 min, 30% B; 6 min, 50% B; 7 min, 70% B; 10 min, 80% B; 12 min, 100% B; 14 min, 100% B; 14.2 min, 5% B and 16 min, 5% B. The flow rate was 0.35 mL/min. The injection volume was 5 μL. The mass range was from *m*/*z* 100 to 1200. The resolution was set at 70,000 for the full MS scans, and 17,500 for HCD MS/MS scans. The collision energy was set at 10, 20, and 40 eV. The mass spectrometer operated as follows: spray voltage, 3800 V (+) and 3200 V (−); sheath gas flow rate, 40 arbitrary units; auxiliary gas flow rate, 15 arbitrary units; capillary temperature, 320 °C; auxiliary gas heater temperature, 350 °C; S-lens RF level, 55. All RAW-formatted output files from LC-MS/MS were processed using software Progenesis QI V2.3 (Nonlinear, Dynamics, Newcastle, UK) for baseline filtering, peak identification, integral, retention time correction, peak alignment, and normalization [27]. The main parameters of 5 ppm precursor tolerance, 10 ppm product tolerance, and 5% product ion threshold were applied. Compound identification was based on a precise mass-to-charge ratio (*m*/*z*), secondary fragments, and isotopic distribution using The Human Metabolome Database (HMDB), Lipidmaps (V2.3), Metlin, and self-built databases. The extracted data were then further processed by removing any peaks with a missing value (ion intensity = 0) in more than 50% of the groups, by replacing the zero value with half of the minimum value, and by screening according to the qualitative results of the compound. Compounds with resulting scores of below 36 (out of 60) points were also deemed to be inaccurate, and were removed. A data matrix was combined from the positive and negative ion data.

The matrix was imported into R to carry out principal component analysis (PCA) to observe the overall distribution of the metabolites among the samples and the stability of the entire analysis process. Orthogonal partial least-squares-discriminant analysis (OPLS-DA) and partial least-squares-discriminant analysis (PLS-DA) were utilized to distinguish the metabolites that vary between groups. To prevent overfitting, seven-fold cross-validation and 200 response permutation testing (RPT) were used to evaluate the quality of the model. The variable importance of projection (VIP) values obtained from the OPLS-DA model were used to rank the overall contribution of each variable to group discrimination. A two-tailed Student’s *t*-test was further used to verify whether the differences in metabolites between groups were significant. Differential metabolites were selected, with VIP values of greater than 1.0, and *p*-values of less than 0.05. The KEGG (https://www.kegg.jp/, accessed on 27 December 2022) database was used for the pathway enrichment analysis.

### 2.9. Statistical Analysis

The data presented herein are expressed as the mean ± standard deviation. The SPSS program (version 16.0) (SPSS, Inc., Chicago, IL, USA) was used for statistical analyses, and *p* < 0.05 was used as the threshold. All experiments were performed in triplicate.

## 3. Results

### 3.1. Sequence Analyses of Aoaar

**Figure 1:**
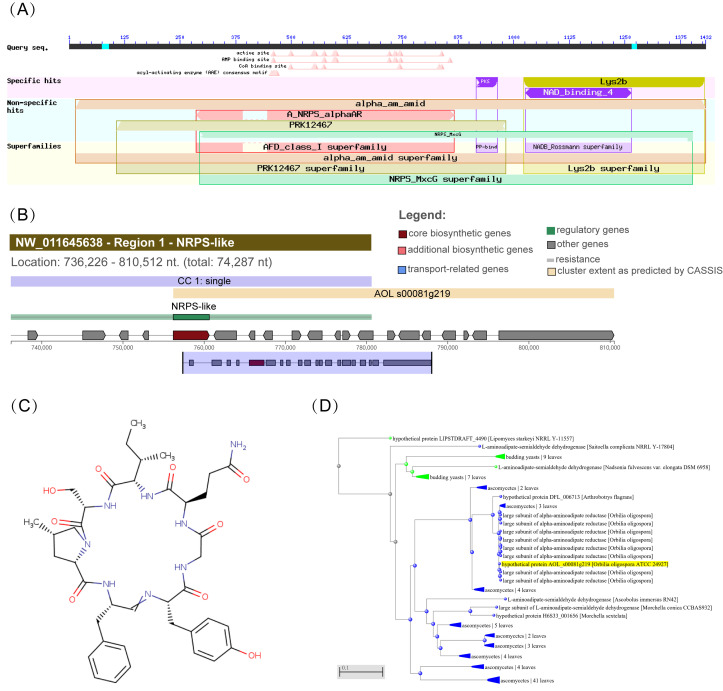
Sequence and phylogenetic analyses of *Aoaar*. (**A**) Conserved domains of *Aoaar* from Blastp prediction using amino acid sequence; antiSMASH-predicted biosynthetic gene clusters (NRPS) (**B**) and their predicted core structures (**C**); (**D**) Phylogenetic tree of gene *Aoaar*.

In this study, the predicted functions of *Aoaar* were identified based on domain analysis, AntiSMASH, and phylogenetic analysis. The analysis of the amino acid sequence encoded by *Aoarr* revealed that *Aoaar* includes at least six component domains (Figure 1A), in keeping with other LYS2 orthologs encoding α-aminoadipate reductases (EC 1.2.1.31). Residues 15–1432 constitute an *alpha*_am_amid domain (accession: TIGR03443), residues 286–866 comprise an A_NRPS_*alpha*AR domain (accession: cd17647), residues 1023–1429 comprise a thioester reductase domain (accession:COG3320), residues 10–982 comprise a peptide synthase domain (PRK12467), residues 1026–1275 comprise a NAD_binding_4 domain (accession: pfam07993), and residues 916–964 comprise a phosphopantetheine attachment site (accession: smart00823). Based on the analysis of the conserved domains, we inferred that the function of *Aoaar* was related to lysine synthesis as α-aminoadipate reductase, and non-ribosomal peptide (NRP) synthesis as non-ribosomal polypeptide synthase (NRPS). We next proceeded with AntiSMASH and phylogenetic analysis. The AntiSMASH predictions indicated that a total of 19 secondary metabolite-producing gene clusters were identified. The *Aoaar* gene was predicted to be the core gene in a NRP biosynthetic gene cluster containing an AMP-binding (278–743 AA), a PCP (900–967 AA), and an NAD_binding_4 (1025–1274 AA) that is necessary for NRP biosynthesis in *A.oligospora* (Figure 1B). The core peptide representing a putative NRP was also predicted, as shown in Figure 1C (Nostocyclopeptide A2) [28]. A phylogenetic tree of *Aoaar* genes was constructed using MEGA7.0 software, and the results also support the interpretation that *Aoaar* has a high degree of similarity to α-aminoadipate reductases (Figure 1D). Whole genome annotation indicates that the *Aoaar* gene was the only gene encoding α-aminoadipate reductase in *A. oligospora*. In summary, the bioinformatics analyses indicate that *Aoaar* is a multifunctional gene that is mainly involved in primary metabolism for lysine biosynthesis, and secondarily, acting in polypeptide biosynthesis.

### 3.2. The Disruption of Aoaar Affects the Growth and Nematocidal Activity of A. oligospora

**Figure 2:**
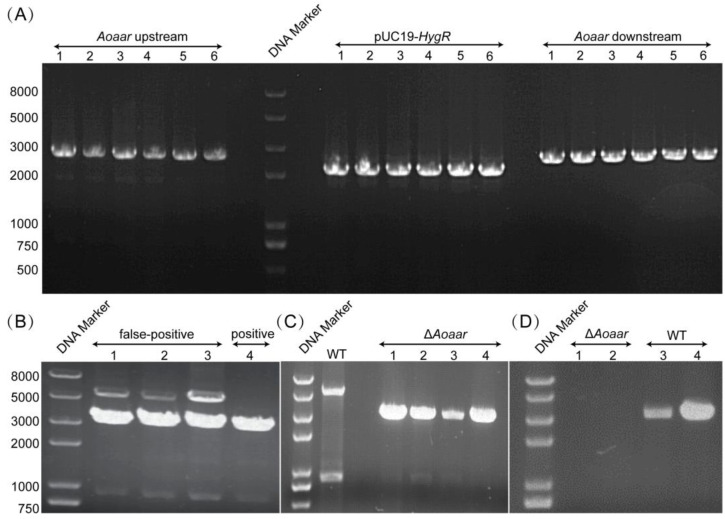
Screening and verification of Δ*Aoaar* mutant strain. (**A**) Verification of the PCR amplification of the *Aoaar* upstream homologous arm (2500 bp), downstream homologous arm (2500 bp), and hygromycin resistance gene expression cassette (2121 bp); (**B**) Verification of the Δ*Aoaar* mutant strain using diagnostic PCR, showing only the predicted 3145 bp of the amplicon. As a comparison, both the 3145 bp and 5438 bp fragments were generated for false-positive transformants; (**C**) Verification of four positive Δ*Aoaar* mutant strains, and the WT strain that was used as control; (**D**) RT-PCR verification: the WT strain has a target band (4299 bp), and the Δ*Aoaar* mutant strain has no target band via RT-PCR.

To understand the effects of the *Aoaar* gene on the growth and physiology of *A. oligospora*, the *Aoaar* gene was knocked out via homologous recombination (Figure 2 and Figure S1). Specifically, the *Aoaar* upstream and downstream sequences (each 2500 bp) were successfully cloned from the genomic DNA and marked as *Aoaar*-up and *Aoaar*-down (Figure 2A). The hygromycin resistance expression cassette was also successfully cloned from the pUC57–*HygR* plasmid and marked as *hygR* (Figure 2A). The three amplicons (*Aoaar*-up, *hygR*, and *Aoaar*-down) were ligated to the linearized pUC19 (*NdeI* and *PciI*) plasmid, followed by screening on ampicillin-supplemented LB plates and verification via DNA sequencing. The linearized homologous recombinant (HR) fragment containing the upstream and downstream sequences (2500 bp each), and the hygromycin resistance cassette was then amplified from the knockout plasmid. After protoplast transformation using the HR fragment, and hygromycin screening on double-layer TB3 agar plates, positive transformants were obtained. Figure 2B shows that three false positive transformants (both 3145 and 5438 bp amplicons were generated) and one positive transformant (a 3145 bp fragment was the only fragment generated) were obtained. We also compared the fragment-sized differences between the WT strains and four positive transformants. Only 5438 bp amplicons were generated in the WT strains, and only the 3145 bp fragment was generated in positive transformants (Figure 2C). We further verified the knockout strains at the mRNA level using RT-PCR. The results showed that the WT strain had a target band (4299 bp), and that the Δ*Aoaar* strain had no target band. We concluded that a Δ*Aoaar* knockout strain was successfully obtained through homologous recombination, and that *Aoaar* gene expression was disrupted (Figure 2).

**Figure 3:**
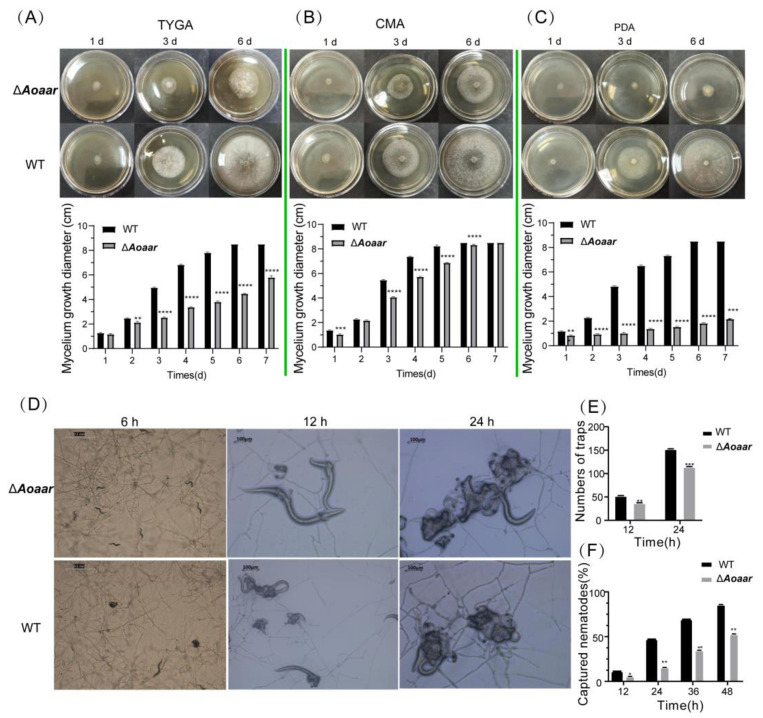
Comparison of growth, trap formation, and nematocidal activity between the WT strain and Δ*Aoaar* mutants. Mycelial growth rates of the WT strain and Δ*Aoaar* mutants incubated on TYGA (**A**), CMA (**B**), and PDA (**C**) media. (**D**) Trap formation of the WT strain and mutants, as induced by addition of nematodes for 6, 12, and 24 h, based on microscopic observation. (**E**) The number of nematode-induced traps produced by the WT and mutants at 12 and 24 h. (**F**) Percentage of nematodes captured by the WT strain and mutants at various time-points. *p* < 0.05 (*), *p* < 0.01 (**), *p* < 0.001 (***), *p* < 0.0001 (****).

The WT and Δ*Aoaar* strains were compared for growth ability, spore germination, and the predation of nematodes. The results showed that the growth rate of the Δ*Aoaar* strain decreased by 40–60% compared with the WT on TYGA, TG, and PDA media (Figure 3A–C). It is worth noting that the Δ*Aoaar* strain almost completely lost the ability to grow in PDA medium (Figure 3C). The conidial number of the WT and Δ*Aoaar* strains on CMA medium was counted, with the results showing that the WT strain generates a 6.2 × 10^5^ spores/mL suspension, which was significantly higher than that of the Δ*Aoaar* strain (2.7 × 10^5^ spores/mL) (Figure S2A). In addition, the spore germination rates of WT and the Δ*Aoaar* strain were determined in WA medium, with the results indicating that the knockout of *Aoaar* significantly impaired spore germination by *A. oligospora* (Figure S2B). Importantly, our study found that *Aoaar* was relevant to the ability of *A. oligospora* to prey on nematodes (Figure 3D). The *Aoaar* knockout significantly destroyed the formation of the traps of *A. oligospora* in the WA plates, both at 12 h and 24 h, following the addition of *C. elegans* (*p* < 0.05) (Figure 3E). The *Aoaar* knockout resulted in a significant decrease to varying degrees in the capture rate of *A. oligospora* to nematodes, in the period of 12 to 48 h after the addition of *C. elegans* (Figure 3F).

### 3.3. Disruption of Aoaar Reprogrammed Amino Acid-Related Primary and Secondary Metabolism

The above results show that *Aoaar* had a significant effect on the growth, spore germination, and predation of nematodes (Figure 3 and Figure S2). Sequence analysis suggests that *Aoaar* is involved in several areas of primary and secondary metabolism. To explore the potential mechanisms by which knockout of *Aoarr* brings about multiple changes to the phenotype of *A. oligospora*, the global metabolic differences between WT and Δ*Aoaar* strains on PDA media were compared via metabolomic analysis.

**Figure 4:**
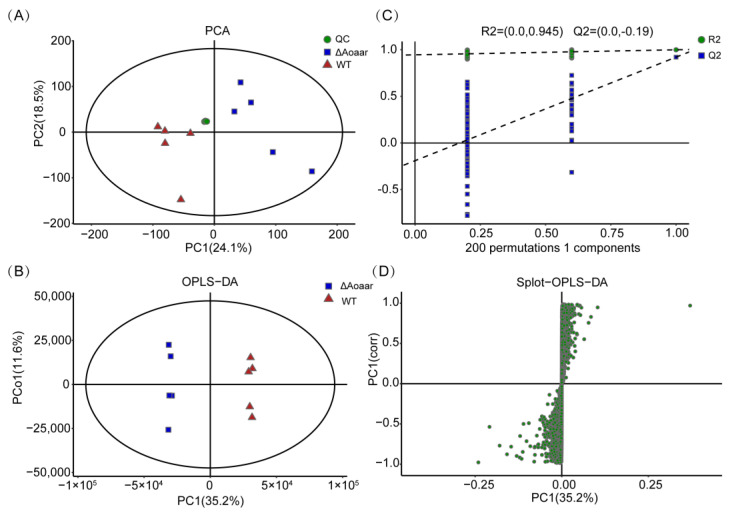
Multivariate analysis of metabolomics data matrix. (**A**) Principal component analysis (PCA). (**B**) Orthogonal partial least squares discriminant analysis (OPLS-DA). (**C**) The permutation plot of OPLS-DA. (**D**) S-plot of OPLS-DA.

UPLC-Q Exactive Orbitrap/MS was used for the metabolomics analysis, and the repeatability and reliability of the data were assessed using PCA and OPLS-DA, and found to be high. As shown in Figure 4A,B, the *Aoaar* knockout had discernible effects on the *A. oligospora* metabolome. To avoid overfitting of the supervised PLS-DA model, the permutation test for PLS-DA was applied, and the results indicated that the PLS-DA model was valid (Figure 4C). An S-plot displays the distribution of metabolites between the WT and the Δ*Aoaar* strains (Figure 4D).

**Figure 5:**
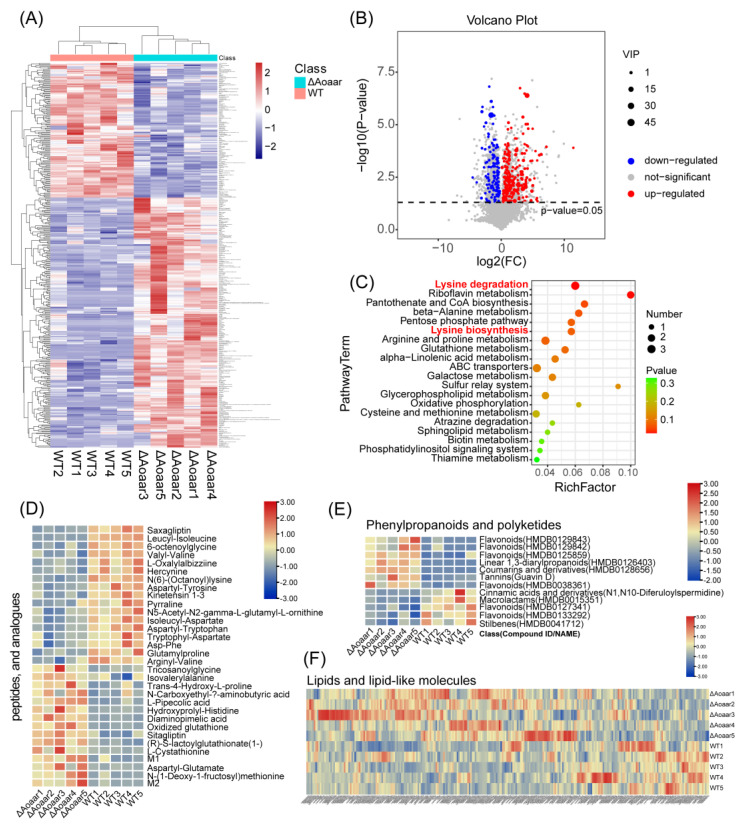
Global metabolic changes between WT and the Δ*Aoaar* mutant. (**A**) The global metabolite changes are represented as a heatmap. (**B**) Metabolite changes are represented as a volcano plot depicting significant metabolite changes between WT and the *Aoaar* knockout strain (fold change > 2, *p* < 0.05, and VIP value > 1). (**C**) Pathway enrichment analysis. (**D**) The differences in peptides and analogues between WT and the Δ*Aoaar* mutant. (**E**) The differences in phenylpropanoids and polyketides between WT and the Δ*Aoaar* mutant; (**F**) The differences in lipids and lipid-like molecules between WT and the Δ*Aoaar* mutant.

The relative abundance of all the identified metabolites was illustrated using a heatmap (Figure 5A). The heatmap shows that the *Aoaar* gene knockout resulted in a global metabolic reprogramming in *A. oligospora*. Among the 8339 annotated metabolites (SM2), more metabolites were increased after the *Aoaar* knockout. The differential metabolite analysis results are shown by the volcano plot in Figure 5B. Based on the differential metabolite analysis, 380 metabolites were significantly changed (fold change > 2, *p* < 0.05, and VIP value > 1) after the *Aoaar* knockout. Compared with the WT strain, 133 metabolites decreased, and 247 increased in the *Aoaar* knockout strain. Pathway enrichment analysis was undertaken using the differential metabolites identified above, and the results showed that the *Aoaar* knockout mainly influenced the pathways, including amino acid metabolism (lysine biosynthesis and degradation, beta-alanine metabolism, arginine and proline metabolism, glutathione metabolism, and others) and lipid metabolism (*alpha*-linolenic acid, glycerophospholipid metabolism, sphingolipid metabolism, and phosphatidylinositol signaling system). Lysine metabolism was especially significantly changed (Figure 5C). In addition, carbon metabolic pathways, such as the pentose phosphate pathway and galactose metabolism, and oxidative stress-related metabolism such as glutathione metabolism, were also changed (Figure 5C). Specifically, the abundance of peptides and analogues (Figure 5D), and phenylpropanoids and polyketides (Figure 5E), and lipids and lipid-like molecules (Figure 5F) were significantly different between the WT and the Δ*Aoaar* strain. We noted that the numbers of peptides and analogues (Figure 5D), and of phenylpropanoids and polyketides (Figure 5E) that were significantly different from the WT were either upregulated or downregulated in roughly equal numbers in the *Aoaar* knockout, whereas the majority of lipids and lipid-like molecules were increased in the *Aoaar* mutation (Figure 5E). Metabolomics analysis confirmed the results of sequence analysis at the metabolite level, with *Aoaar* playing a key role in both lysine metabolism and secondary metabolism, such as NRP synthesis and the production of polyketides, and even lipid metabolism.

## 4. Discussion

The present study characterized the orthologous α-aminoadipate reductase (*Aoaar*) in *A. oligospora* through sequence analysis, phenotypic assessments, and metabolomic analyses. Sequence analysis indicated that the gene of *AOL_s00081g219* not only encodes α-aminoadipate reductase involved in lysine biosynthesis, but it is also a core gene of the secondary metabolite non-ribosomal peptide (NRP) biosynthesis gene cluster. This is consistent with results that have been obtained in other fungi. In *Candida albicans*, the *Caaar* gene has been shown to be homologous to non-ribosomal peptide synthetases in the amino-terminal two-thirds of the protein [29]. An analysis of the sequences of *Schizosaccharomyces pombe* and *Penicillium chrysogenum* also showed that the putative protein encoded by *aar* shares strong homology with the peptide antibiotic synthetases [30,31]. The AAR enzymes provide the catalytic activity to reduce α-aminoadipic acid into its semialdehyde, and to display an A-T-R domain line-up (a typical domain layout of a non-ribosomal peptide synthetase) [32]. Thus, in the present study, a non-proteinogenic amino acid that serves as a monomer for peptidic natural products made via NRPSs was found for the first time in the nematode-trapping fungus *A. oligospora*. That means that the knockout *Aoaar* disrupts both lysine-related metabolism and secondary metabolism such as non-ribosomal peptide biosynthesis in *A. oligospora*.

**Figure 6:**
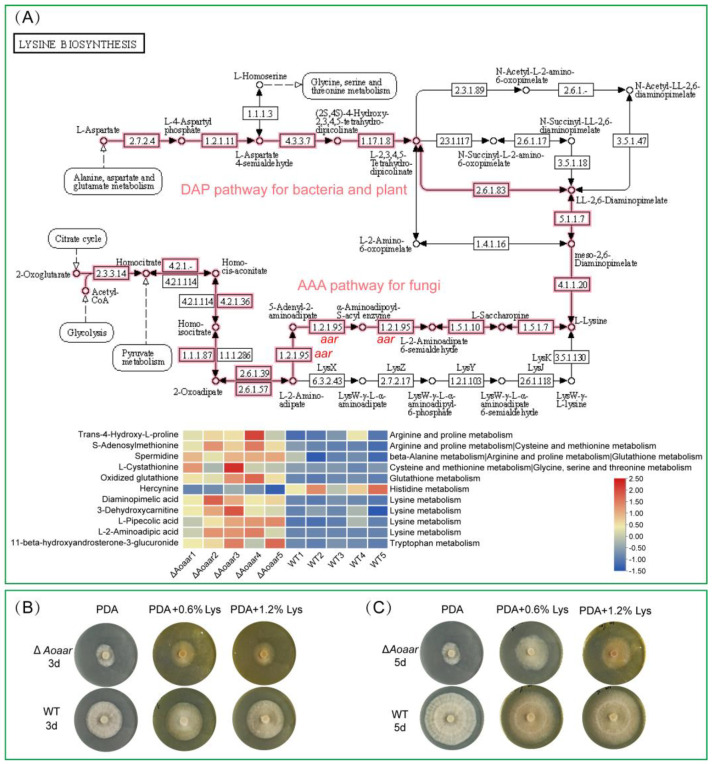
The effect of *Aoaar* knockout on the lysine biosynthesis pathway. (**A**) Diagram showing pathways for lysine biosynthesis in bacteria and plants (DAP pathway), and in fungi (AAA pathway). The heatmap shows the abundance of metabolites (left) involved in amino acid-related metabolic pathways (right). (**B**) A comparison of the effects of lysine supplementation on the growth of WT and the *Aoaar* knockout strain at day 3. (**C**) A comparison of the effects of lysine supplementation on the growth of WT and the *Aoaar* knockout strain at day 5. aar, α-aminoadipate reductase.

The phenotypic assessments indicated that the knockout of *Aoaar* significantly impeded the growth, trap formation, and nematocidal activity of *A. oligospora*. Therefore, we hypothesize that lysine biosynthesis and non-ribosomal peptide biosynthesis are involved in regulating growth, trap formation, and the nematocidal activity of *A. oligospora*. This hypothesis was further strengthened by our metabolomics analysis. Based on the metabolomics analyses, lysine metabolism was shown to be the most significant metabolic pathway affected in the knockout mutant. Based on the pathway enrichment analysis, L-2-aminoadipic acid, which is the substrate of *Aoaar*, accumulated significantly in the *Aoaar* mutant strain, compared with the WT strains (Figure 6). Although NRP-related secondary metabolic pathways were not found among the pathways most significantly impacted (Top 20) (Figure 5), an analysis of the secondary metabolites in the identified differential metabolites indicated that the biosynthesis of peptides and analogues (Figure 5D) was considerably disturbed via the *Aoaar* knockout. In addition, the phenylpropanoids and polyketides (Figure 5E), and the lipids and lipid-like molecules (Figure 5F) were also significantly altered in abundance between the WT and the Δ*Aoaar* strains. Summarizing, we demonstrated that the effects of *Aoaar* on both the lysine biosynthesis pathway and the secondary metabolism result in the phenotypic differences between the *Aoaar* mutant strain and the WT strain in the present study. On one hand, α-aminoadipate reductase as the core gene of the NRPS biosynthesis gene cluster can be directly involved in the biosynthesis of secondary metabolites. On the other hand, there are numerous examples of fungal alkaloids or peptides that have lysine as a structural element or biosynthetic precursor. There are also cases where aminoadipate pathway intermediates are incorporated into secondary metabolites [14,32,33]. In the present study, metabolites in the amino acid metabolism-related pathways were significantly changed in the *Aoaar* mutant strain (Figure 6). This leads to global perturbations on the primary and secondary metabolic pathways, and results in changes in the growth, trap formation, and nematocidal activity of *A. oligospora*.

The results from some -omics analyses indicated that amino acid metabolism remodeling plays a critical role during the transition from vegetative hyphae to trap cells. A study by Yang et al. indicated that the *Aoras2* and *Aorheb* mutants produced fewer traps, and that their extracellular proteolytic activities were significantly lower than those of the WT strain. Multi-omics analyses of the nematode-trapping fungus *A. oligospora* indicated that cellular amino acid metabolism mediated the effects that *Aoras2* and *Aorheb* had on mycelial growth and on changes in conidiation, stress resistance, and predacious ability [34].

The roles of secondary metabolites on mycelial growth, conidiation, stress resistance, and predacious ability are supported by a considerable body of research [35,36,37,38,39,40,41]. Non-ribosomal peptides (NRP) have also been discussed in a previous study. A metabolomics study on 100 wild isolates of nematode-trapping fungi in three different species found that several small peptides (<1.5 kDa), such as the non-ribosomal peptide desferriferrichrome, notably increased in abundance as the fungi switched lifestyle to the predatory stage [42].

In order to verify the role of lysine biosynthesis on the growth and development of mycelium in *A. oligospora*, different levels of lysine were added to the PDA medium to survey the growth and development difference between the *Aoaar* knockout mutant strain and the WT strain (Figure 6). Our results indicated that the growth and development of *Aoaar* mutant strain were significantly recovered when lysine was added. This further supports the results from our metabolomics analysis. In addition, we found that although amino acid supplementation reduced the difference in growth between the mutant strain and the wild type, it still grows slower than the WT strain, suggesting the existence of other mechanisms to explain the effect of the *Aoaar* knockout on the growth and development of mycelium, rather than its only effects being on lysine metabolism. This may be related to effects of *Aoarr* on the intermediates of the lysine metabolism pathways and on NRP biosynthesis. In the present study, we found that lysine biosynthesis and NRP biosynthesis were coupled by the gene *Aoaar*. This provides a molecular basis for the understanding of the relationship between amino acid metabolism, the synthesis of the secondary metabolite NRP, and the biocontrol possibilities of nematode-predator fungi.

The α-aminoadipate (AAA) pathway is unique to fungi, and is thus a potential target for the rational design of biocontrol agents based on the nematode-trapping fungi, and for the control of the nematode-trapping fungi (Figure 6A). The successful colonization of biocontrol strains in the complex and stable soil microenvironment is an important difficulty in the large-scale application of biocontrol agents, and overcoming this challenge is the key to achieving nematode biocontrol [38,43]. In the present study, we found that *Aoarr* is required for the growth and predatory activity of *A. oligospora*; therefore, we can potentially enhance lysine biosynthesis by increasing the expression of *Aoarr*, and by supplementing the metabolic intermediates of the fungi-specific AAA pathway and secondary metabolic pathways associated with peptide synthesis. This may confer a potential growth advantage on the strain, and favor colonization. In addition, due to lysine biosynthesis being a biochemical difference between higher fungi, bacteria, and plants, the α-aminoadipate pathway for lysine biosynthesis offers a unique opportunity to develop specific molecular probes for the detection, imaging, and control of biocontrol strains, and as an effective research tool [44,45]. From this perspective, the present study provides new strategies for the development of biocontrol agents, and tools for the further study of nematode predation fungi.

## 5. Conclusions

The present study characterized the *A. oligospora* α-aminoadipate reductase ortholog of *aar* (*Aoaar*) through comparative sequence analysis, and via phenotypic and metabolomic analyses of the knockout mutant. *Aoaar* resembles Lys2-type α-aminoadipic acid reductases, which serve in fungal L-lysine biosynthesis. Our study proved the important positive effects of lysine biosynthesis on the mycelial growth, conidiation, stress resistance, and predacious abilities of the nematode-trapping fungus *A. oligospora* for the first time, at the molecular level. Our sequence analysis indicated that *Aoaar* also features an A-T-R domain arrangement, which was relevant for the biosynthesis of small natural product non-ribosomal peptides (NRPs) in *A. oligospora*. Although the results of our metabolomic analysis are limited in supporting the role of *Aoaar* in mycelial growth through the regulation of NRP synthesis, the lysine supplementation test suggests that *Aoaar* knockdown has other regulatory mechanisms besides affecting mycelial growth through lysine synthesis; the role of *Aoarr* for NRP biosynthesis, and the relationship between *Aoarr*, lysine metabolism, NRP biosynthesis, and mycelial growth and predatory activity are the focus of subsequent studies. This study provides an important reference for uncovering the role of amino acid metabolism in nematode capture by nematode-trapping fungi, and likewise for clarifying the biological functions of multifunctional genes using metabolomics techniques.

## Data Availability

Not applicable.

## Supplementary Materials

The following supporting information can be downloaded at: https://www.mdpi.com/article/10.3390/jof9020206/s1, Supplementary Material S1 (SM1) (WORD): Table S1. Primer sequences used in this study; Figure S1. Knockout of gene *Aoaar* via homologous recombination technology; Figure S2. Disruption of *Aoaar* (*g219*) impaired the spore generation and germination of *A. oligospora*. Supplementary Material S2 (SM2) (EXCEL): Metabolomics results, including annotated metabolites and differential metabolites.
