# Supplementary material for: Roles of the Fungal-Specific Lysine Biosynthetic Pathway in the Nematode-Trapping Fungus Arthrobotrys oligospora Identified through Metabolomics Analyses
Source: J Fungi (Basel). 2023 Feb 5;9(2):206. doi: 10.3390/jof9020206 (PMC9963897; doi:10.3390/jof9020206)
Supplement: Supplementary file 1 [file jof-09-00206-s001.zip › Supplementary material 1ú║Supplementary tables and figures.pdf]

Article

Roles of the Fungal-Specific Lysine Biosynthetic Pathway in the Nematode-Trapping Fungus *Arthrobotrys oligospora* Identified through Metabolomics Analyses

Hengqian Lu [1,2,†], Shuai Wang [1,2,†], Tiantian Gu [1,2], Liangyin Sun [1,2] and Yongzhong Wang [1,2,3,*]

1 School of Life Sciences, Anhui University, Hefei 230601, China; hengqianlu@ahu.edu.cn (H.L.); w2980077322@163.com (S.W.); gu042666@163.com (T.G.); slyanki@foxmail.com (L.S.)
2 Key Laboratory of Human Microenvironment and Precision Medicine of Anhui Higher Education Institutes, Anhui University, Hefei 230601, China
3 Anhui Key Laboratory of Modern Biomanufacturing, Hefei 230601, China
* Correspondence: yzwang@ahu.edu.cn; Tel.: +86-187-8886-3053
† These authors contributed equally to this work.

Table S1. Primer sequences used in this study.

Primers	Sequence (5′-3′)	Fragment size
Aoaar-1F	5'tgctggccttttgctcacatgtaaaaggaggaggaggaagaa gaggag-3'	2500 bp
Aoaar-1R	5'ATCATCTTCTGtgtgttttgactattgattgattaattgat tgaaggcaa-3'	
Hyg-F	5'tcaaaacacaCAGAAGATGATATTGAAGGA GCATTTTTGGG-3'	2121 bp
Hyg-R	5'ccatagttttcAAAGAAGGATTACCTCTAAA CAAGTGTACCTG-3'	
Aoaar-3F	5'ATCCTTCTTTgaaaactatgggctagcttttatggaatg ag-3'	2500 bp
Aoaar-3R	5'ttgtactgagagtgcaccatatgtacggggaacaaatggaaatt ggtgg-3'	
g219-Dx-F	5'-gaggtccacaatgccttggat-3'	3145bp（M）
g219-Dx-R	5'-tttgactctccgtaagcctaagac-3'	5438bp（WT）

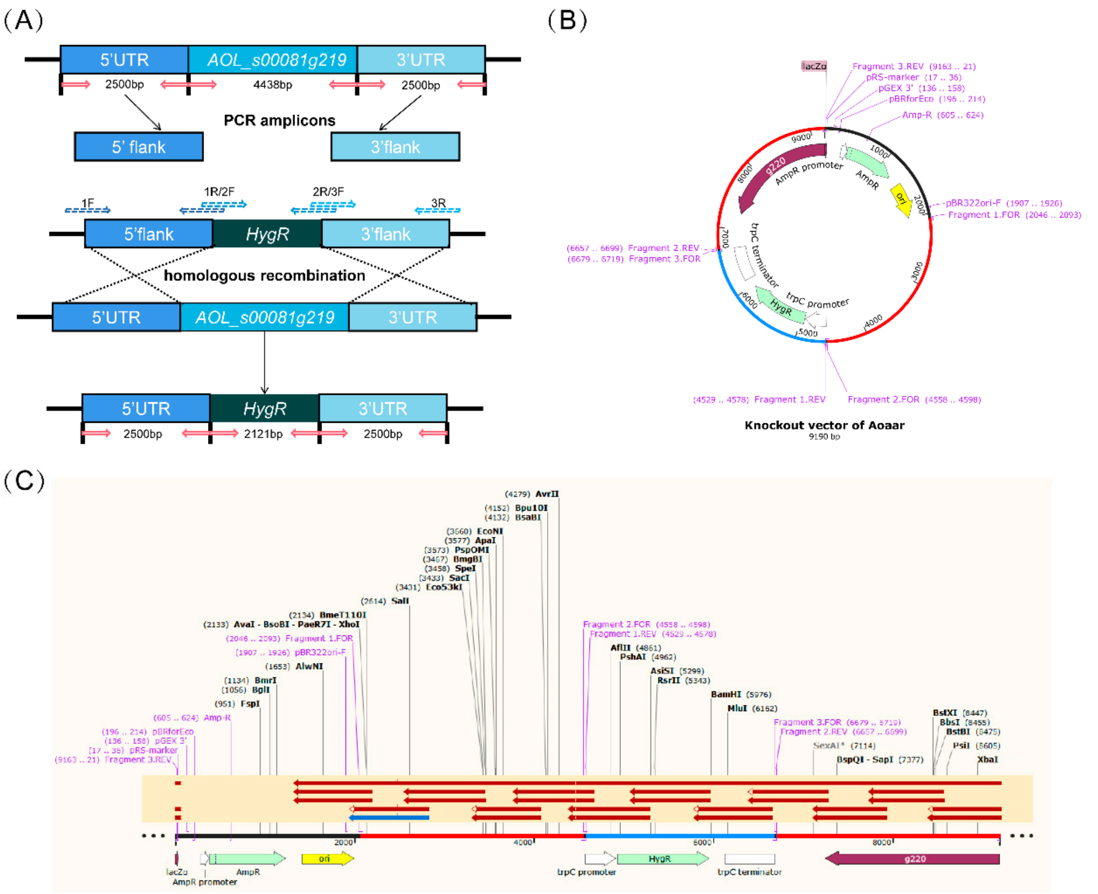

Figure S1. Knockout of gene *Aoaar* by homologous recombination technology. (A) The principle of Aog207 knockout by homologous recombination; **(B)** Knockout vector of *Aoaar*; **(C)** sequencing results of positive transformants of *A. oligospora*.

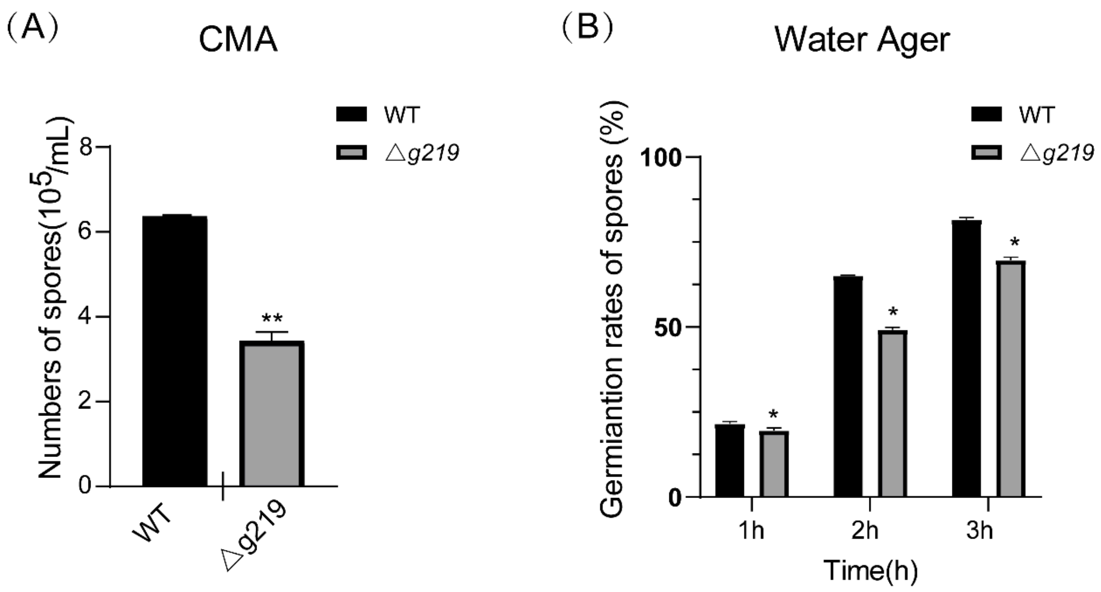

Figure S2. Disruption of *Aoaar* (g219) impaired the spore generation and germination of *A. oligospora*. (A) The difference of conidial number between WT and ΔAoaar strain; (B) The difference of the spore germination rate between WT and ΔAoaar strain.
